# Supplementary material for: Assessment of crop damage by rodent pests from experimental barley crop fields in Farta District, South Gondar, Ethiopia
Source: PLoS One. 2021 Aug 12;16(8):e0255372. doi: 10.1371/journal.pone.0255372 (PMC8360600; doi:10.1371/journal.pone.0255372)
Supplement: Attachment — Submitted filename: Response to Reviewers.docx [file pone.0255372.s001.docx]

**Responses to reviewers**

- Page 4, 2^nd^ paragraph, last line- the sentence “Permit for this work was obtained from south Gondar Zone Environmental Protection and Land Administration Bureau” added.
- Acknowledgment –the sentence –“ We would like to thank Addis Ababa University for financial support” deleted
- Please keep the sentence “‘the author(s) received no specific funding for this work.” As it is under Funding Statement
- Manuscript has been amended according to Plos One writing style requirement.

Q1. **Apart from rodents, will there be other species that have caused damage to the crops in this area? Such as birds, insects, etc.? If insects can also cause damage to crops, is the control area enclosed by barbed wire not rigorous?**

**Answer**: Yes, animals other than rodents like birds and insects can damage the crops. Birds during sowing (before ploughing) can pick the seeds and cause damages during maturation stage. But the sample plots were kept from birds by the researcher and field assistant farmers as it is explained in the methodology part of the manuscript (see methodology part marked in red (page 5 para 1, last three lines)). Moreover, birds do not damage seedling and boosting stages of the barley crops. So the researchers have considered the effects of birds on the experimental barley crop field during sowing and maturation periods.

Insects can also take the barley seeds during sowing (before ploughing). This taking of seeds takes place in both treatment and control unites as the control unites were excluded from rodents by fine wire mesh after ploughing. So there is no any effect on the result. However, after ploughing, the damage is negligible as insects do not dig and pick the barley seeds before germination as rodents do. But during sowing stage (before ploughing) the damage by insects was for both control and treatment units because the plots were excluded by fine wire mesh after ploughing. In addition, to increase its reliability, the researchers took 5 samples by considering the different parts (edge and middle) of each selected 40 by 40 m sample farmlands.

Q2**. The author compared the crop damage caused by rodents in 2019 and 2020, but in fact, climatic conditions may also cause damage to crop harvests. Has the author considered the impact of climatic conditions on crop harvests during the past two years of harvest? Also, is there a difference in climate in areas far away from forests and near forests?**

**Answer**: Yes, the climate condition may not be the same for the two cropping years (2019 and 2020). But, the intention of the experiment was to see the amount of yield variations between 2 by 2 m control and treatment plots. So the environment may equally affect both the treatment and control units equally unlike rodents damage. That means, the comparison of the yield was not simply taken as the yield variation between the two cropping years of the same plots. In addition, the experiment was taken on the same place and crop for two cropping years. So the climate variation may not have effect on the overall result. In addition to this, The distance between farmland near the forest and away from the forest is less than 500 m, may not have climatic variation. The aim of comparison of crop damage by rodents near the forest and away from the forest was to show the effect of the forest on the surrounding farmland crops hence the forest serves as a shelter for rodents. We conclude this fact from our finding.

Q3. **The author selected 2 areas (close to the forest and far from the forest), a total of 4 plots were selected for research. The overall study area is very large, so I think the selected sample plot (40×40m) a little small, and the authors selected 2 sample plot for each area (close to the forest and far from the forest), is the number of the sample plot for each area a little less?**

**Answer:** Yes, the researchers selected two 40 X 40 m plots of lands near the forest and the other two far from the forest. For each 40 X 40 m plots of lands, we sampled four 2 X 2 m control plots, 8 near the forest and the other 8 away from the forest (16 in total) for the experiment. In addition, for direct observation (in seed, seedling boosting and maturation stage damage assessments), five (5) 1 X 1 m samples for each 40 by 40 m plots of lands (20 in total) were taken. This helped the researcher to manage all samples efficiently for two successive cropping years. There is also homogeneity of sample areas in terms of crop grown and topography of the land, in this regard the sample size is enough for the study.

Q4. Figures in the manuscript are not numbered

Answer: There are two figures in the manuscript and indicated in the text as Fig 1 (page 4, paragraph 1, line 3 and Fig 2 page 5 2^nd^ paragraph, line 7).
